# The C.O.S.T.A.S. project (Clinically Oriented Spasmolysis Treatment- A Scorescale): a pilot study

**DOI:** 10.1016/j.bas.2026.106053

**Published:** 2026-04-15

**Authors:** Konstantinos Lintas, Xenia Winter, Robert Sarge, Emanuele Maragno, Oliver Müller

**Affiliations:** Department of Neurosurgery, Dortmund Hospital/ University Witten/Herdecke, Muensterstraße 240, 44145, Dortmund, Germany

**Keywords:** Subarachnoid hemorrhage, Cerebral vasospasm, Intra-arterial spasmolysis, Scoring system, Artificial intelligence

## Abstract

**Introduction:**

Symptomatic cerebral vasospasm (CVS) following aneurysmal subarachnoid hemorrhage (SAH) presents a significant therapeutic dilemma. Intra-arterial spasmolysis is a potentially life-saving intervention, associated with procedural risks.

**Research question:**

The aim of C.O.S.T.A.S. (Clinically Oriented Spasmolysis Treatment – A Score Scale) project is to develop a clinically based, patient-oriented scoring system, to identify patients with SAH who may benefit from intra-arterial spasmolysis for CVS.

**Material and methods:**

Parameters and clinical data from SAH-patients were categorized and weighted according to their clinical relevance creating the scoring-system. Parameters included are age, smoking, intracranial arterial atherosclerosis, body-mass index, renal function, mechanical ventilation metrics, noradrenaline requirements, and an artificial intelligence (AI)-based vasospasm-risk prediction model. Each parameter was assigned a score ranging from 0 to 3 points, with a maximum cumulative score of 18.

**Results:**

The scale provides a comprehensive overview of each patient's clinical status. It integrates data from artificial intelligence–based model, along with other relevant factors, identifying patients at higher risk of developing CVS who may benefit from intra-arterial spasmolysis. In addition, it serves as a triage tool by efficiently assessing the patient's condition and determining their suitability for invasive intervention, balancing the potential risks and benefits of the procedure.

**Discussion and conclusion:**

The C.O.S.T.A.S. project is the first scoring-scale specifically designed to guide intra-arterial spasmolysis in SAH patients with CVS. While currently in prototype form, it offers a practical, transparent, and adaptable framework for individualized treatment decisions. A multicenter application, testing, and indexing are crucial for achieving maximal classification accuracy and parameter weighting.

## Highlights

• Clinically based, patient-oriented scoring system.
• Each parameter a score from 0 to 3 points, with a maximum cumulative score of 18.
• Identifying patients at risk of CVS who may benefit from intra-arterial spasmolysis.
• Assessing the patient's condition, determining suitability for intervention.

## 1 Introduction

Subarachnoid hemorrhage (SAH), even though a benign disease, comes with deleterious sequelae in about 30% for the patients afflicted. For the repair of the aneurysm, either a surgical approach is employed, necessitating to prepare the guarding vessel and the aneurysm itself, sometimes making even a temporary clipping or ventricular overpacing necessary. Or, an endovascular procedure of aneurysm obliteration is preferred, what can be likewise an arduous job when proceeding the guide wire and catheters beyond the bifurcation of the internal carotid artery (ICA) (Ihn et al., 2018).

Vasospasms are one of the most common and maybe the most life-threatening conditions following the rupture of a cerebral aneurysm. Over the years various studies argued that the occurrence of vasospasms determines the outcome of the treatment and could be predicted on the base of e.g. the clinical condition, bleeding patterns on cerebral computed tomography etc. at the time of the admission of these patients (Wilson et al., 2012; Frontera et al., 2006; Haedo et al., 2023).

On the one hand, intra-arterial spasmolysis offers an effective treatment after the occurrence of vasospasms. Studies have shown that intra-arterial spasmolysis using nimodipine infusion was associated with low treatment-specific complications and offers an independent life to an extensive number of patients (Vossen et al., 2023), (Santos et al., 2022).

On the other hand, numerous neurointensive clinics embrace the idea, that an invasive endovascular intervention as a rescue therapy for patients effected by vasospasms, leads not to a better outcome than only inducing a conservative one. This objective is supported by a few studies and publications, in particular, when taking into consideration the risks related with the intra-arterial spasmolysis. Although most of these publications mention that only a subgroup of patients benefits from the combination of both treatments, they cannot determine nor recognise these individuals (Güresir et al., 2022), (Vatter et al., 2022).

However, all grading scales, studies, and medical practices are deficient in applying the patient's unique baseline situation, incorporating data of various qualities: clinical, demographic, neurointensive treatment, lab chemistry, etc. Every patient brings a variety of different co-variables along with their medical record that likely necessitate individualizing their therapy in a specific way.

The aim of this study is to create and propose a score-scale that assesses the capability of patients for invasive intra-arterial spasmolysis, who are suffering from cerebral vasospasm based on their clinical condition at a holistic level. An easily applied grading system offers a more individualized and patient-oriented treatment plan, which aligns perfectly with today's requirements for a patient-centred healthcare philosophy.

## 2 Materials and methods

### Ethical approval

The use of data was approved by the ethic committee of the medical association of Westfalen-Lippe (2021-687-f-S). The study was carried out under the guidelines of Good Clinical Practice according to the WMA principles of the declaration of Helsinki.

### 2.1 Definition of symptomatic vasospasm/delayed cerebral ischemia

A clinical deterioration caused by delayed cerebral ischemia (DCI) is defined as the occurrence of focal neurological impairment (such as hemiparesis, aphasia, apraxia, hemianopsia, or neglect), or a decrease of at least 2 points on the Glasgow Coma Scale (either on the total score or on one of its individual components [eye, motor on either side, verbal]). This should last for at least 1 h, is not apparent immediately after aneurysm occlusion, and cannot be attributed to other causes by means of clinical assessment, CT or MRI scanning of the brain, and appropriate laboratory studies. The occurrence of clinical deterioration caused by DCI should be described separately from the results of angiography. The term cerebral vasospasm should be reserved for angiographic arterial narrowing of a radiological test (either CT angiography, MR angiography or digital subtraction angiography) (Frontera et al., 2009),(Suwatcharangkoon et al., 2019), (Vergouwen et al., 2010).

### 2.2 Diagnostic and treatment of symptomatic vasospasm/delayed cerebral ischemia

All patients at our institute were routinely monitored by neurologic examination and Doppler examination. Symptomatic cerebral vasospasm was suspected following an acceleration of the cerebral blood flow that either transcended >190 cm/s or increased by a **Δ** of at least 50% of the measurement before. In cases of uncertainty a CT-perfusion scan was complemented. In a suspected event of vasospasm, induced hypertension with a mean arterial pressure (MAP) of 80-90 mmHg, depending on the clinical condition and the comorbidities of each patient, is induced by fluid resuscitation and noradrenalin, if necessary. For verification, a digital subtraction angiography (DSA) was performed with subsequent intra-arterial spasmolysis, which was applied to all patients of both cohorts, treated surgically and endovascularly. If necessary, multiple interventions were done with additional balloon-angioplasty in short proximal stenosis. Permissive hypertension with MAP >100 mmHg was maintained, if vasospasms persisted.

### 2.3 Score-scale parameters

The purpose of the study was to create a comprehensive and easy-to-use score-scale that integrates all relevant aspects of the current neurological and overall clinical status of each patient suffering from cerebral vasospasms. The multitude of clinical parameters, which combined can determine the suitability of a patient for intra-arterial spasmolysis at any given moment, necessitates a thorough medical assessment. In our framework, the criteria applied, while fundamental and perhaps seemingly insufficient, incorporate all the essential information needed for a thorough evaluation.

• Age

From a chronological viewpoint, medical treatment of the elderly (geriatrics) starts from the age of 65. Older adults often experience several aging-related common clinical conditions that increase vulnerability to morbidity and poor outcomes. Aging is associated with an increase in comorbidities and a higher risk of “multimorbidity” or the co-occurrence of two or more chronic conditions, which include hypertension, diabetes, chronic obstructive pulmonary disease, heart failure, cancer, and cognitive impairment (Arai et al., 2012).

Cerebral blood flow declines, leading to impaired oxygen delivery, slowed metabolism, and altered activity and production of neurotransmitters. Further, endothelial cells lose function, and the blood–brain barrier becomes more permeable, exposing older adults to increased risk to the central nervous system from systemic insults. Respiratory system changes with aging increase vulnerability to pulmonary infections, respiratory failure and mucociliary transport becomes dysfunctional (Brunker et al., 2023). There is also a decrease in renal mass due to loss of renal cortex, the effective renal blood flow decreases up to 10% per decade of life and there is a variable decrease in glomerular filtration rate (GFR) with age, and many GFR calculations do not account for the physiologic changes of aging.

• Cigarette smoking

Cigarette smoking is a major health hazard, with 5.4 million premature deaths worldwide every year and an average loss of 13 to 15 years of life expectancy. Smoking is established as one of the most critical risk factors for subarachnoid hemorrhage. There is a complex yet interesting interplay between cigarette smoking exposure, vascular inflammation, and cerebral aneurysm formation and rupture (Chalouhi et al., 2012).

It goes without saying, that cigarette smoking has a big influence on the physiology of the lungs, and in extension on the invasive mechanical ventilation of such patients. Studies have shown that during the intubation period heart rate; systolic, diastolic, mean arterial pressure and rate-pressure product showed more pronounced fluctuations in smokers than in non-smokers (Nikota et al., 2011).

• Intracranial arterial vasosclerosis

Intracranial atherosclerosis (ICAS) is a major cause of strokes worldwide. Luminal stenosis of atherosclerotic vessels directly leads to insufficient blood supplying at downstream vessel segments. Intracranial arteries exhibit some unique histological features that are quite different from those of comparatively sized vessels elsewhere in the body: a denser internal elastic lamina, a thinner media with slight development of elastic fibers, and a less abundant adventitia with decreased elastic fibers and without external elastic lamina (Wang et al., 2018).

Intracranial arterial atherosclerosis was defined in our study protocol as ≥50% stenosis in an intracranial vessel. Studies have demonstrated that this degree of vessel narrowing can be reliably detected with high sensitivity across various diagnostic methods such as a radiological test (CT angiography, MR angiography or digital subtraction angiography), transcranial doppler (TCD) and/or intraoperatively (Yang et al., 2017), (Banerjee and Chimowitz, 2017).

After verification of CVS, a digital subtraction angiography was performed with subsequent intra-arterial spasmolysis with nomodipin and/or balloon-angioplasty in short proximal stenosis. Vasosclerosis directly leads to insufficient blood supply in the downstream vessel segments, exacerbating the existing clinical deterioration caused by delayed cerebral ischemia. Furthermore, atherosclerotic plaques and their frequently detected calcification create rigid vessels that are prone to stenosis, reducing the ability for vasodilation and occasionally preventing it altogether.

• Weight and BMI Index

Obesity is a pandemic of the twenty-first century. Studies showed that obesity may have less effects on the outcome of patients with mainly cranial neurosurgical diseases (Ali and Prabhakar, 2016). Nonetheless it has a significant impact on various organ-systems of the body and thus needs a well-planned neurointensive management. Obese patients with multiple co-morbidities like type 2 diabetes, hypertension, and cardiovascular disease, are expected to have more complications than normal individuals. Obesity may influence the risk of aneurysm formation and rupture and/or the outcome of patients who have aneurysmal subarachnoid hemorrhage.

Among those medical challenges, are difficult airway management by generalised accumulation of adipose tissue, especially around the soft palate, tongue, and hypopharyngeal region causing narrowing; non-reliability of electrocardiography and noninvasive blood pressure; difficulty of inserting intravenous access and central venous cannulation due to thick subcutaneous layer of fat; medication dosing; patient transport etc.

• Glomerular Filtration Rate (GFR)

After the verification of symptomatic cerebral vasospasms, a digital subtraction angiography with subsequent intra-arterial spasmolysis using nimodipin was indicated.

Contrast-induced acute kidney injury (CI-AKI), formerly termed contrast-induced nephropathy (CIN), describes an association between intravenous or intra-arterial contrast administration and renal impairment. The American College of Radiology introduced new terminologies to clarify the causal relationship between the use of intravascular contrast and the development of acute kidney injury:

• contrast-induced acute kidney injury (CI-AKI): acute kidney injury caused by contrast administration
• contrast-associated acute kidney injury (CA-AKI) (postcontrast acute kidney injury (PC-AKI)): acute kidney injury due to any cause within 48 h after contrast administration as an association (Contrast-induced acute kidney injury).

Estimated glomerular filtration rate (eGFR) has been used for the assessment of renal function before intravenous contrast injection. This is calculated from the patient's age, race, sex and serum creatinine level. The use if this value in our score system is of a great importance.

• Invasive Mechanical Ventilation

Mechanical ventilation or assisted ventilation is the medical term for using a machine called a ventilator to fully or partially provide artificial ventilation.

There is a variety of parameters for a patient on a ventilation machine. In this study we included two of them; partial pressure of oxygen (PaO2)/fraction of inspired oxygen (FiO2) (PaO2/FiO2 (P/F)) ratio and positive end-expiratory pressure (PEEP).

The fraction of inspired oxygen (FiO2) is the concentration of oxygen in the gas mixture. It is an estimation of the oxygen content a person inhales and is thus involved in gas exchange at the alveolar level.

In the setting of critical patients, FiO2 is routinely used to assess the lungs' capacity for gas exchange, using the PaO2/FiO2 (P/F) ratio, where PaO2 represents the partial pressure of oxygen.

Positive end-expiratory pressure (PEEP) is the positive pressure that will remain in the airways at the end of the respiratory cycle (end of exhalation) that is greater than the atmospheric pressure in mechanically ventilated patients. By opening up airways, the alveolar surface increases, creating more areas for gas exchange and somewhat improving ventilation.

• Katecholamine/noradrenalin

Noradrenaline is a naturally occurring sympathomimetic which is an agonist on alpha1, alpha2 and beta1 receptors and has little or no effect on beta2 or dopamine receptors. Noradrenaline raises systolic and diastolic blood pressure via alpha effects causing peripheral vasoconstriction, which may reduce blood flow in the kidneys, liver and skeletal muscle. It is being applied to increase blood pressure in acute, severe, hypotensive states when low systemic vascular resistance persists despite adequate fluid resuscitation (Norepinephrine dosage and administration),(Safer Care Victoria. Noradrenaline (norepinephrine), 2018). In our institution it is administered as continuous intravenous infusion through a central access line via infusion pump. In this study, the use and dosage of noradrenaline have binary importance. First, as mentioned above, it has a catalytic effect on the induced hypertension in patients suffering from cerebral vasospasms and is the first step in their non-invasive treatment. On the other hand, the dosage administered to a patient is globally considered a direct indicator of their clinical status and reflects clinical stability. Given that digital subtraction angiography with subsequent intra-arterial spasmolysis is a time-consuming, invasive procedure conducted in a non-ICU environment, noradrenaline therapy is a decisive parameter.

• Artificial-intelligent prediction model of occurrence of cerebral vasospasms based on machine-learning

Certainly, in a study of symptomatic cerebral vasospasms, evidence-based observations such as the clinical condition and bleeding patterns on cerebral computed tomography at the time of admission should not be overlooked. Foundational grading scales (Fisher, Hunt and Hess, and BNI), alongside key demographic characteristics (e.g., age, sex, comorbidities), and specific details of early treatment (including operative duration, type of intervention, and intraoperative aneurysm rupture), were integrated in a comprehensive and systematic manner.

At our institution, we developed a customized, practice-oriented artificial intelligence algorithm designed to estimate the individual probability of developing symptomatic vasospasm, enablying the implementation of personalized therapeutic protocols based on each patient's unique risk profile (Lintas et al., 2025).

Eighty-seven patients with aneurysmal SAH during a 24 months-period of time were included for study purposes (45 surgical & 42 endovascular). The Holdout and the Cross-Validation method was used to evaluate the above-mentioned model. In addition, we decided to use the Support Vector Machines (ksvm) classification method, as after various checks it was evident that its results showed a higher accuracy than the other ones tested. The R programming environment was used to create a "user-friendly" command line in order to be able to prognosticate the occurrence of vasospasms.

## 3 Results

**Fig. 1:**
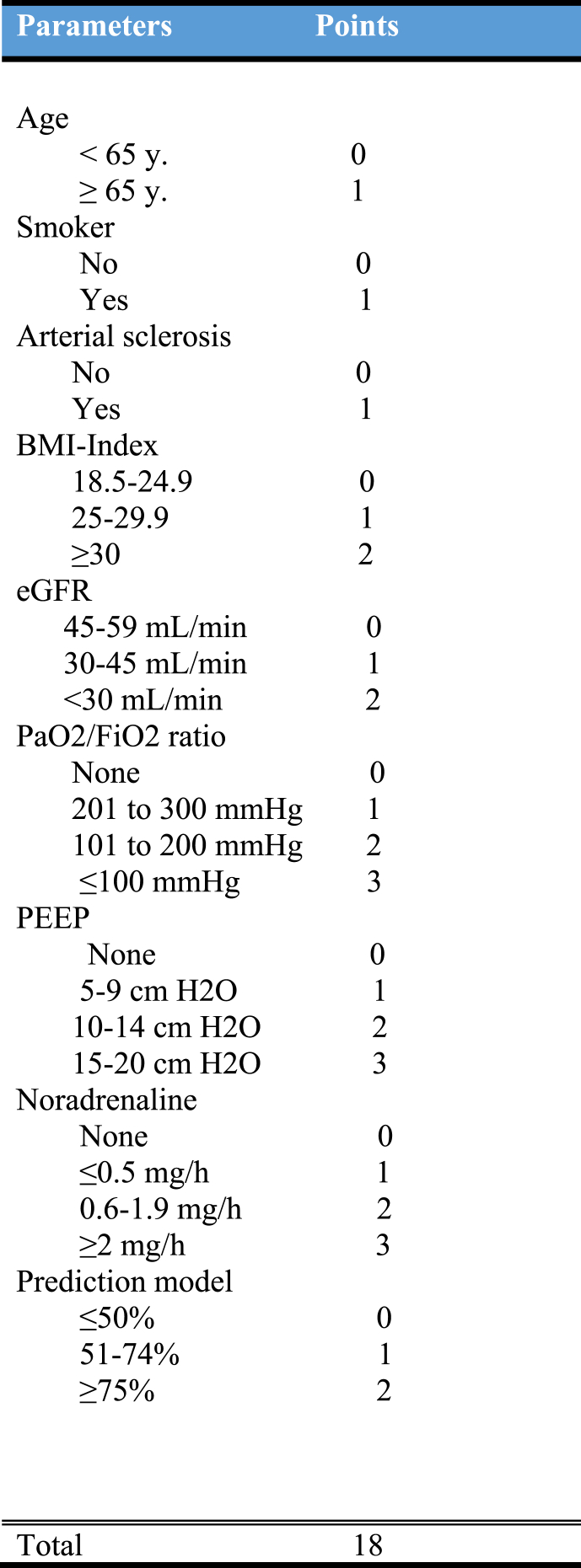
Patient-oriented score-scale for intra-arterial spasmolysis.

After detailed analysis and interdisciplinary consultation, we designed the first clinical and patient-oriented score-scale for intra-arterial spasmolysis following the occurrence of symptomatic cerebral vasospasms due to subarachnoid hemorrhage (Fig. 1).

Each parameter was assigned a grade point. Given their varying importance to the clinical status of a patient, the grading scale ranges from one to three points. The maximum total score sums up to 18 points.

The age parameter was divided into two groups, awarding patients ≥65 years old one point. The same principle applies to patients who smoke cigarettes, as well as those who show signs of intracranial arterial atherosclerosis (IAA) ≥50% in a radiological test (CT angiography, MR angiography or digital subtraction angiography), transcranial doppler (TCD) and/or intraoperatively. Each criterion met is given one point.

In order to include the weight aspects of a clinical subject, we decided to employ the globally accepted BMI Index, considering its widespread use and easy application. Therefore, we the patients into three groups: *Normal weight* (18∙5-24∙9) with zero points, *Overweight* (25-29∙9) with one point, and *Obesity* (≥30) with two points (National Heart et al.).

According to the Kidney Disease Outcome Quality Initiative (KDOQI), the estimated glomerular filtration rate (eGFR) thresholds led to a categorization of patient cohorts into three groups: low risk (45-59 mL/min) with zero points, moderate risk (30-45 mL/min) with one point, and high risk (<30 mL/min) with two points (American Kidney Fund).

The mechanical ventilation coefficient was determined using two parameters: the partial pressure of oxygen (PaO2) divided by inspired oxygen (FiO2) ratio (PaO2/FiO2 (P/F) ratio and positive end-expiratory pressure (PEEP). The PaO2/FiO2 ratio was categorized according to the Berlin criteria for ARDS into three groups: mild (201 to 300 mmHg) with one point, moderate (101 to 200 mmHg) with two points, and severe (less than 100 mmHg) with three points (ARDS Definition Task Force et al., 2012).

Similarly, the PEEP factor was divided into three groups: mild (5-9 cm H2O) with one point, moderate (10-14 cm H2O) with two points, and severe (15-20 cm H2O) with three points.

Given that recruitment manoeuvres, which should be used within the context of lung protection and not merely as a mean of improving oxygenation, we included both parameters in our score scale (Hess, 2015).

The administration, dosage, metric system, and severity index of the catecholamine/noradrenaline factor in the equation is a very sensitive and difficult matter to define. Lacking a universal metric system, different countries and continents use non-identical units to describe the dosage of each medication given to a patient (Malinowski et al., 2008).

Although vasopressor requirements are widely recognized as a surrogate marker of hemodynamic instability and shock severity, there is currently no universally accepted or validated classification system that stratifies patients based on norepinephrine dose alone. Thus, the grading system we propose may be controversially criticized and denounced.

Existing scoring systems, such as the Sequential Organ Failure Assessment (SOFA), incorporate vasopressor use within the cardiovascular component, where escalating norepinephrine requirements are directly associated with increasing severity of circulatory failure(Reinikainen et al., 2025).

Recent literature has increasingly emphasized the prognostic value of norepinephrine dosing. Observational studies and meta-analyses demonstrate a clear dose–response relationship between norepinephrine requirements and mortality, supporting its role as a continuous marker of disease severity. In parallel, contemporary investigations have attempted to define pragmatic dose thresholds for clinical use. These studies suggest approximate ranges for mild (<0.1 μg/kg/min), moderate (0.1–0.3 μg/kg/min), severe (0.3–0.5 μg/kg/min), and refractory shock (>0.5–1.0 μg/kg/min), although these cutoffs remain empirical and lack universal consensus. (Cortes et al., 2025),(Pölkki et al., 2024).

The therapeutic goal of catecholamine (norepinephrine) administration was to achieve a mean arterial pressure (MAP) ≥ 80 mmHg. Accordingly, the norepinephrine requirements of each patient were assessed in relation to this target. For standardization within our institution, the administered dosages were converted into mg/h, reflecting local clinical practice.

To ensure full transparency, we will describe how catecholamine use is managed at our institution. Noradrenaline is the catecholamine of choice in our hospital. It is administered via Syringe Unit/Pump IV infusion through a central venous catheter (CVC) or midline. A large peripheral vein (antecubital or proximal) may be used only in an emergency and when central access is planned. We use noradrenaline 5 mg/5 mL vials to prepare the infusion. These 5 mL of the vial are diluted to 50 mL with 45 mL of 0∙9% sodium chloride (NaCl) in a luer lock syringe. The end product is a 50 mL luer lock syringe, with a concentration of 0∙1 mg/mL.

Despite the complexity of the dosing, we decided to subcategorize the patients into four groups according to their noradrenaline needs: no noradrenaline with zero points, low risk (≤0∙5 mg/h) with one-point, moderate risk (0∙6-1∙9 mg/h) with two points, and high risk (≥2 mg/h) with three points.

Another controversial parameter of our score scale is our artificial-intelligent prediction model of occurrence of cerebral vasospasms based on machine-learning. The patient data used for the statistical analysis and in the creation of this model, along with their subdivisions, included sex, age, H&H scale, Fisher scale, BNI scale, comorbidities (arterial hypertension, cardiovascular diseases, pulmonary diseases, none), anticoagulation (acetylsalicylic acid (ASA), direct oral anticoagulants (DOAC), antiplatelet medication, combination, none), and duration of the operation or intervention. It demonstrated accuracy currently ranges from 61% to 86%.

This analysis reflects the initial performance of the model at implementation. Owing to its AI-based architecture, its predictive accuracy is expected to improve with the integration of larger clinical datasets and will be further refined through prospective multicenter validation. Standardized protocols are currently in development.

In addition, the weighting coefficients assigned to individual variables and their respective subgroups should be derived through rigorous statistical approaches, including meta-analytical methods, and incorporated into the predictive framework. Such refinement is essential to enhance model robustness, improve generalizability, and minimize statistical bias.

Our predictive model, although unique and still in its early stages, can categorize patients into ‘high risk’ and ‘low risk’ cases, considering all the aforementioned factors. This helps identify patients more likely to suffer from CVS and who will consequently benefit from invasive therapy.

We clustered the probability predicted by the AI model into three categories and scored them accordingly: low risk (≤50%) with zero points, moderate risk (51-74%) with one point, and high risk (≥75%) with two points.

## 4 Discussion

This is the first patient-centred and clinically oriented score-scale offering a holistic view of patients suffering from cerebral vasospasms. The implications of applying this grading system are twofold. First, it integrates data from the artificial intelligence prediction model, age, pack years, and other factors, to identify patients who are more likely to suffer from cerebral vasospasms and who, consequently, will benefit from invasive intra-arterial spasmolysis. Second, it serves as a patient triage tool, efficiently describing the clinical status of a patient and assessing their suitability for invasive treatment, thereby balancing the complications and advantages of this procedure.

Cerebral vasospasms and their invasive intra-arterial treatment are highly controversial topics in the neurosurgical field. This dichotomy and incongruity stem from the invasive nature of the treatment, the associated complications, and the clinical diversity of patients suffering from cerebral vasospasms. As previously mentioned, the majority of studies investigating this condition suggest that a subgroup of patients may benefit from a combination of invasive and non-invasive treatments, but they fail to accurately identify these individuals.

The aim of the C.O.S.T.A.S. project was to address this issue. Given the wide spectrum and multitude of clinical aspects, parameters, and values characterizing each patient, it is unrealistic and unfeasible to incorporate them all. The criteria included in our score-scale offer a relatively precise, convenient and user-friendly holistic view of the patient, despite appearing elementary and deficient.

Several grading systems have been developed to assess the severity of subarachnoid hemorrhage and to estimate the risk of subsequent cerebral vasospasm; however, none were specifically designed to guide therapeutic decision-making for vasospasm or intra-arterial spasmolysis. Radiological classifications such as the Fisher Scale and the Modified Fisher Scale primarily quantify the extent and distribution of subarachnoid blood and serve as indirect predictors of vasospasm risk rather than its severity. Clinical grading systems, including the World Federation of Neurosurgical Societies (WFNS) Scale and the Hunt and Hess (H&H) Scale, reflect the neurological condition at presentation but do not incorporate dynamic physiological or radiographic changes occurring during the vasospasm phase. More integrative approaches, such as VASOGRADE, combine clinical and radiological parameters to stratify vasospasm risk; however, they remain limited to prognostic assessment and are not intended to guide interventional management.

In clinical practice, the diagnosis and monitoring of cerebral vasospasm rely on a combination of imaging findings, transcranial Doppler measurements—often interpreted using indices such as the Lindegaard Ratio—and the occurrence of delayed neurological deficits, commonly summarized under the concept of Delayed Cerebral Ischemia (DCI) criteria. Despite this multimodal approach, therapeutic decision-making, particularly regarding the indication and timing of intra-arterial spasmolysis, remains largely subjective and dependent on institutional protocols and individual clinical judgment.

To date, no standardized or validated scoring system exists that integrates clinical status, hemodynamic parameters, imaging findings, and treatment response to guide patient selection for invasive therapies such as intra-arterial spasmolysis. This lack of structured decision-making tools represents a significant gap in the current literature. The scoring model proposed in this study aims to address this limitation by providing a comprehensive, clinically oriented framework for the assessment and stratification of patients with cerebral vasospasm, thereby supporting more objective and reproducible therapeutic decisions.

Certainly, the score-scale can and will be strongly debated and contradicted, as every medical institution uses various clinical standards, divergent scales, and treatment methods, evaluating patients based on different clinical aspects.

The same principle applies to the categorization of our parameters and the point system employed in the design of the score scale. Excluding the "easy-to-handle" parameters like age, weight, and GFR, whose metric gradients are globally accepted, the rest can be easily disputed. The lack of validated and sanctioned from the medical community classification and indexing along with empirical assessments and prototype categorisations for study purposes, provides the foundation for dispute and conflict in the medical field.

Despite the wide array of clinical grading scales now routinely used in neurosurgery, many of these tools began as bold propositions—rooted in abstract concepts and shaped by years of clinical experience. Over time, through multicenter validation, these initially simple scales have proven to be among the most valuable tools in clinical decision-making, significantly reducing the risk of misjudgment and improving patient outcomes (Hemphill et al., 2001), (Sembill et al., 2021).

As the field of neurosurgery continues to advance, the therapeutic landscape is undergoing continuous transformation. Each year, novel technologies and techniques challenge established paradigms, expanding the boundaries of what was previously considered unattainable. In this context, the development of structured, individualized decision-making tools—such as scoring systems that personalize therapeutic strategies—represents a critical step forward. Such approaches should not be confined to a single domain but extended across the broader spectrum of neurotrauma and stroke care, where patient heterogeneity demands precision-guided intervention.

Recent advances in the understanding of secondary mechanisms of neurotrauma have demonstrated that delayed brain injury following subarachnoid hemorrhage is not a uniform phenomenon, but rather the result of complex and interacting pathophysiological pathways, including neuroinflammation, oxidative stress, blood–brain barrier disruption, and microcirculatory dysfunction (Aghili-Mehrizi et al., 2022). These heterogeneous and dynamic processes provide a mechanistic basis for the observed variability in clinical outcomes among patients with seemingly similar initial presentations, thereby reinforcing the need for individualized risk stratification and tailored therapeutic strategies. In parallel, emerging therapeutic approaches, including nanoparticle-based drug delivery systems, exemplify the ongoing shift toward precision medicine in neurovascular disorders, enabling targeted modulation of pathological processes with enhanced spatial and temporal specificity (Hey et al., 2025).

Within this evolving framework, the COSTAS score is designed to integrate demographic, clinical, and physiological parameters to identify patients most likely to benefit from intra-arterial spasmolysis, thereby aligning treatment selection with underlying disease mechanisms. By bridging insights from secondary injury cascades with advances in targeted therapeutic delivery, the COSTAS model represents a translational step toward personalized management of cerebral vasospasm following subarachnoid hemorrhage. In this way, neurosurgical practice moves from a paradigm of “one-size-fits-all” therapy toward a model that emphasizes precision—not only in targeting pathological processes, but also in selecting the right patient for the right intervention at the right time.

## 5 Conclusions

Clinical grading scales play a crucial role in the evaluation and management of patients by standardizing both assessment and subsequent treatment.

This pilot study represents an initial step toward developing and refining our scoring scale. Our scoring model, though currently in a prototype phase, is both unique and innovative, designed to evaluate a patient's suitability for invasive intra-arterial spasmolysis by taking into account their comprehensive clinical status. The model's accessibility, practicality, and straightforward parameters make it a valuable tool for addressing this longstanding clinical challenge. A multicenter application, testing, and indexing are crucial for achieving maximal classification accuracy.

## 6 Limitations

Our study has several limitations. A primary limitation is its monocentric and interdisciplinary design. Many of the included parameters are open to debate and interpretation. For example, a high body mass index (BMI) may reflect increased muscle mass in well-trained individuals rather than obesity. The noradrenaline parameter lacks a standardized severity index based on dosage, necessitating a prototype-based categorization. Similar limitations apply to parameters related to invasive ventilation. With respect to the AI prediction model, its performance is expected to improve with the integration of additional cases and will benefit from broader, multicentric validation. Furthermore, although the command-line interface is designed to be user-friendly, it currently requires familiarity with the specified statistical programming environment.

## Ethical considerations

The use of data was approved by the ethic committee of the medical association of Westfalen-Lippe (2021-687-f-S). The study was carried out under the guidelines of Good Clinical Practice according to the WMA principles of the declaration of Helsinki.

## Author contribution

1. Dr. med. Konstantinos Lintas: data collection, methodology, analysis and evaluation, drafting the manuscript.

2. Xenia Winter: data collection, analysis of results, revising the manuscript.

3. Dr. med. Robert Sarge: senior consultant neurosurgeon, ICU neurosurgery, providing data on the intensive care treated patients, revising the manuscript.

4. Dr. med. Emanuele Maragno: data collection, analysis of results, revising the manuscript.

5. Prof. Dr. med. Oliver Müller: Head Dept. Neurosurgery, drafting the study, methodology, data evaluation, final revision of the manuscript.

All authors read and approved the final version of the manuscript for the submission.

## Data sharing

All data of the present study and the protocol are available after publication upon request from the corresponding author upon reasonable intention to achieve aims in the approved proposal.

## Declaration of generative AI and AI-assisted technologies in the manuscript preparation process

During the preparation of this work, the author(s) used ChatGPT (OpenAI) for the purpose of language refinement, including grammar correction and improvement of academic style. Following the use of this tool, the author(s) carefully reviewed and edited the content as necessary and take full responsibility for the content of the published article.

## Funding statement

This research did not receive any specific grant from funding agencies in the public, commercial, or not-for-profit sectors.

## Declaration of competing interest

The authors declare that they have no known competing financial interests or personal relationships that could have appeared to influence the work reported in this paper.
